# Ferulic Acid on Glucose Dysregulation, Dyslipidemia, and Inflammation in Diet-Induced Obese Rats: An Integrated Study

**DOI:** 10.3390/nu9070675

**Published:** 2017-06-29

**Authors:** Norma Julieta Salazar-López, Humberto Astiazarán-García, Gustavo A. González-Aguilar, Guadalupe Loarca-Piña, Josafat-Marina Ezquerra-Brauer, J. Abraham Domínguez Avila, Maribel Robles-Sánchez

**Affiliations:** 1 Departamento de Investigación y Posgrado en Alimentos, Universidad de Sonora, Blvd. Luis Encinas y Rosales S/N, Colonia Centro, Hermosillo, Sonora, C.P. 83000 Sonora, Mexico; njulietasl@yahoo.es (N.J.S.-L.); ezquerra@guayacan.mx (J.-M.E.-B.); 2 Centro de Investigación en Alimentación y Desarrollo, A.C., Carretera a La Victoria km 0.6, Hermosillo, Sonora, C.P. 83304 Sonora, Mexico; hastiazaran@ciad.mx (H.A.-G.); gustavo@ciad.mx (G.A.G.-A.); abrahamdominguez9@yahoo.com (J.A.D.A.); 3 Departamento de Investigación y Posgrado en Alimentos, Facultad de Química, Universidad Autónoma de Querétaro, Cerro de Las Campanas, S/N, Colonia Las Campanas, Santiago de Querétaro, Querétaro, C.P. 76010 Querétaro, Mexico; loarca@uaq.mx

**Keywords:** ferulic acid, dyslipidemia, inflammation, glucose dysregulation, high fat diet

## Abstract

Obesity is considered to be a low-grade chronic inflammatory process, which is associated with cardiovascular and metabolic diseases. An integral evaluation of the effects of ferulic acid on biomarkers of glucose dysregulation, dyslipidemia, inflammation, and antioxidant potential induced by a high-fat diet (HFD) in rats was carried out. Three groups of male Wistar rats (six per group) consumed a basal diet (BD), which was supplemented with either lard at 310 g/kg (HFD) or lard and ferulic acid at 2 g/kg (HFD + FA), ad libitum for eight weeks. Body weight gain, hyperplasia, and hypertrophy in abdominal fat tissues were higher in the HFD group than in the HFD+FA group. The rats fed a HFD + FA significantly inhibited the increase in plasma lipids and glucose, compared with the HFD group. Biomarkers associated with inflammation were found at higher concentrations in the serum of rats fed a HFD than the HFD + FA group. Plasma antioxidant levels were lower in HFD rats compared to rats fed the HFD + FA. These results suggest that ferulic acid improves the obesogenic status induced by HFD, and we elucidated the integral effects of ferulic acid on a biological system.

## 1. Introduction

The World Health Organization defines overweight and obesity as abnormal or excessive fat accumulation that may impair health [1]. In overweight and obese individuals, the increase in body weight promotes adipose tissue hyperplasia and hypertrophy, which increases secretion of various pro-inflammatory chemokines, cytokines, and hormones, such as C-reactive protein (CRP), resistin, inducible nitric oxide synthase (iNOS), monocyte chemotactic protein (MCP)-1, tumor necrosis factor (TNF)-α, and interleukin (IL)-1, IL-6, and IL-8. This results in a state of low-grade chronic inflammation associated with obesity-related metabolic diseases [2,3]. This condition increases the risk of developing comorbidities, such as coronary artery disease, hypertension, dyslipidemia, insulin resistance, type 2 diabetes mellitus, nonalcoholic fatty liver disease, and respiratory disorders [2,4].

Phytochemicals may have a positive impact on obesity through the control of the lipid metabolism, inflammation, and carbohydrate metabolism [5,6]. Ferulic acid is a hydroxycinnamic acid with potential health benefits due to its antioxidant and anti-inflammatory capacity, among others, and is widely distributed in foods of plant origin [6,7]. In a recent investigation, ferulic acid inhibited the expression of TNF-α and IL-1β in lipopolysaccharide LPS-activated monocyte-derived THP-1 macrophages by inhibiting the activation of nuclear factor-kappa B (NF-κB), which could contribute to preventing chronic inflammatory diseases [7]. In another study, ferulic acid alleviated metabolic syndrome in rats administered high-fat and high-fructose diets [8]. The above results are consistent with Senaphan et al. [9], who showed that ferulic acid alleviates changes in metabolic syndrome in rats through suppression of oxidative stress by down-regulation of p47phox, increased nitric oxide (NO) bioavailability with upregulation of endothelial nitric oxide synthase (eNOS) and suppression of TNF-α [9]. Ferulic acid also prevents acute liver injury by inhibiting intrahepatic inflammation and liver apoptosis in mice via the reduction of hepatocellular degeneration, lymphocyte infiltration, the number of apoptotic hepatocytes, serum levels of tumor necrosis factor alpha (TNF-α) and interferon-γ (IFN-γ), and liver myeloperoxidase (MPO) activity [10].

In the present study, an integral evaluation of the effect of ferulic acid on biomarkers of glucose dysregulation, dyslipidemia, inflammation, and antioxidant potential in rats fed high-fat diets (HFD) was carried out.

## 2. Materials and Methods

### 2.1. Animals and Treatments

Eighteen male Wistar rats (eight weeks old) were obtained from the Department of Research and Postgraduate Studies, Universidad de Sonora (Mexico). They were housed individually in suspended wire mesh-bottomed cages. Lighting in the animal room was on a 12-h light:dark cycle. The temperature was maintained at 22 °C and relative humidity at 40–70%. After a one week acclimation period, rats were randomly assigned to one of the three experimental groups (six per group).

All diets were formulated from the AIN-93G diet [11], with slight modifications. Briefly, to obtain the basal diet (BD), AIN-93G was modified to contain lard and soybean oil at 19 and 24 g/kg of diet, respectively. For induction of obesity, the rats were given a high-fat diet (HFD) (formulated to contain lard and soybean oil at 316 and 32 g/kg of diet, respectively). Ferulic acid (FA) was added to the HFD at 2 g/kg of diet and was referred to as HFD + FA. The composition of the diets is given in Table 1. The level of FA used in the test diet in this study represent moderate, subtoxic and subpharmacologic levels in the diet and were based on levels used in previous studies [12,13]. All diets were stored at 4 °C until fed to the rats. Rats consumed their respective diets and water ad libitum. Body weight and food intake were monitored weekly throughout the experiment. At the end of the eight-week feeding period, rats were anaesthetized using sodium phenobarbital (60 mg/kg). Blood from these rats was drawn from the left atrium of the heart after previous overnight fasting (12 h) and placed into centrifuge tubes coated with and without sodium EDTA. Serum and plasma from each rat were separated by centrifugation (2000 g/15 min), aliquoted in 0.5 mL fractions and stored at −80 °C for later analyses. Abdominal fat was obtained as follows: after opening the peritoneum and removing the viscera, abdominal fat was carefully dissected, weighed and kept in 4% formaldehyde for histological analysis. All animal procedures were conducted in strict conformation with the NIH guidelines [14] for animal care and the study submitted for the approval of the Bioethics Committee of the Research Center in Food and Development (CIAD, A.C.), Hermosillo, Sonora, Mexico (animal permit number: CE/004/2017).

### 2.2. Biochemical Analysis

Plasma glucose, triglycerides (TG), total cholesterol (TC), high-density lipoprotein-cholesterol (HDL-C), and low-density lipoprotein-cholesterol (LDL-C) concentrations were measured with commercial kits, following the supplier’s instructions (Randox, Crumlin, Antrim, UK). Apolipoprotein B (ApoB) and Apolipoprotein A1 (ApoA1) concentrations were quantified with commercial ELISA kits from LifeSpan BioSciences, Inc. (Seattle, WA, USA). Very-low-density lipoprotein-cholesterol (VLDL-C) concentration was calculated by dividing triglycerides by five [15]. The atherogenic index was calculated as (total cholesterol – HDL-C)/HDL-C [16]. Interleukin-1β (IL-1β), interleukin-4 (IL-4), interleukin-6 (IL-6), interleukin-10 (IL-10), interleukin-12 (IL-12), and granulocyte macrophage colony-stimulating factor (GMC-SF) concentrations were measured by commercial ELISA kits from QIAGEN (Germantown, MD, USA). Serum insulin concentration was determined with an ELISA kit (Industrial MexLab SA de CV, Zapopan, Jalisco, México). The homeostatic model assessment index of insulin resistance (HOMA-IR) for each group assayed was calculated by multiplying insulin plasma (μUI/mL) by glucose plasma (mmol/L) and dividing by 22.5 [17].

### 2.3. Antioxidant Capacity

#### 2.3.1. Oxygen Radical Absorbance Capacity Assay (ORAC)

The ORAC assay was carried out on a microplate fluorescence reader FLUOstar^®^ Omega (Ortenberg, Germany). The procedure was based on a previous report by Huang et al. [18]. The experiment was conducted at 37 °C at pH 7.4 with a blank sample in parallel. The analyzer was programmed to record the fluorescence of fluorescein (10 nM) every five minutes after addition of 240 mM AAPH (2,2′-azobis (2-methylpropionamidine) dihydrochloride), for 90 min, at an excitation wavelength of 485 and an emission wavelength of 530 nm. The final results were calculated using the differences between areas under the curve of fluorescein decay of the blank and the sample, and were expressed as micromoles of Trolox equivalents ((±)-6-hydroxy-2,5,7,8-tetramethylchromane-2-carboxylic acid) per mL (μmol TE/mL).

#### 2.3.2. Trolox Equivalent Antioxidant Capacity (TEAC) Assay

Trolox equivalent antioxidant capacity (TEAC) assay is based on the ability of antioxidant molecules to scavenge the ABTS•+ cation radical (2,2′-azino-bis(3-ethylbenzothiazoline-6-sulfonic acid) diammonium salt), which produces a change in color that can be spectrophotometrically quantified [19]. A stable stock solution of ABTS•+ was prepared by mixing 5 mL of an aqueous solution of ABTS (7 mM) with 0.088 mL of sodium persulfate (148 mM), and incubating it in the dark at room temperature for 16–18 h. The ABTS•+ working solution was prepared immediately before use by diluting the stock solution in ethanol (∼1:88, *v*/*v*), and its absorbance was adjusted to 0.700 ± 0.02 at 734 nm. Then, 280 µL of the ABTS•+ working solution was combined with 10 µL of the sample in a microplate well. The changes in absorbance (734 nm) were recorded as ABTS•+ radical-scavenging activity. A standard curve was prepared using Trolox as a standard, which was used to convert the changes in absorbance of the samples to μmol of Trolox equivalents (TE)/mL of plasma.

### 2.4. Histological Analysis

Abdominal fat tissue was extracted, fixed in 10% formalin and paraffin embedded. The tissue of was cut into 3–5 μm sections, which were stained with hematoxylin and eosin (H and E). Adipocyte size (superficial area) and number of adipocytes per area on abdominal fat tissue were evaluated. The adipocyte superficial area was measured with the software ZEN 2 (Carl Zeiss Microscopy GmbH, Göttingen, Germany).

### 2.5. Statistical Analysis

Results are expressed as the mean ± standard error mean (SEM) (*n* = 6). Samples were analyzed in duplicate. To contrast dietary groups, one way analysis of variance (ANOVA) followed by Tukey’s comparison tests was used. The level of significance was *p* < 0.05. Correlations between response variables were calculated using standard Pearson correlation. The statistical software JMP 5.0.1 (SAS Institute, Inc., Cary, NC, USA) was used.

## 3. Results

### 3.1. Effect of FA on Body Weight Gain, Diet Intake, Food Efficiency, and Adipose Tissue Weight

The induction of obesity in rats was achieved by administration of a high-fat diet for 60 days. This was due to the diet composition, as the rats fed the HFD had 50% more caloric content compared to rats fed the BD. The rats fed a HFD showed an increased body weight gain (BWG), energy intake, caloric efficiency, and weight of abdominal fat tissue (WAFT) compared to rats fed the BD (Table 2).

As shown in Table 2, intake of HFD+FA decreased body weight gain (15%, *p* < 0.05) and WAFT values (28%, *p* < 0.05) compared to the HFD group, and similar results were observed with the fat index (23%, *p* < 0.05).

### 3.2. Effect of Ferulic Acid on the Serum Lipid Profile (Biochemical Analysis)

**Figure 1:**
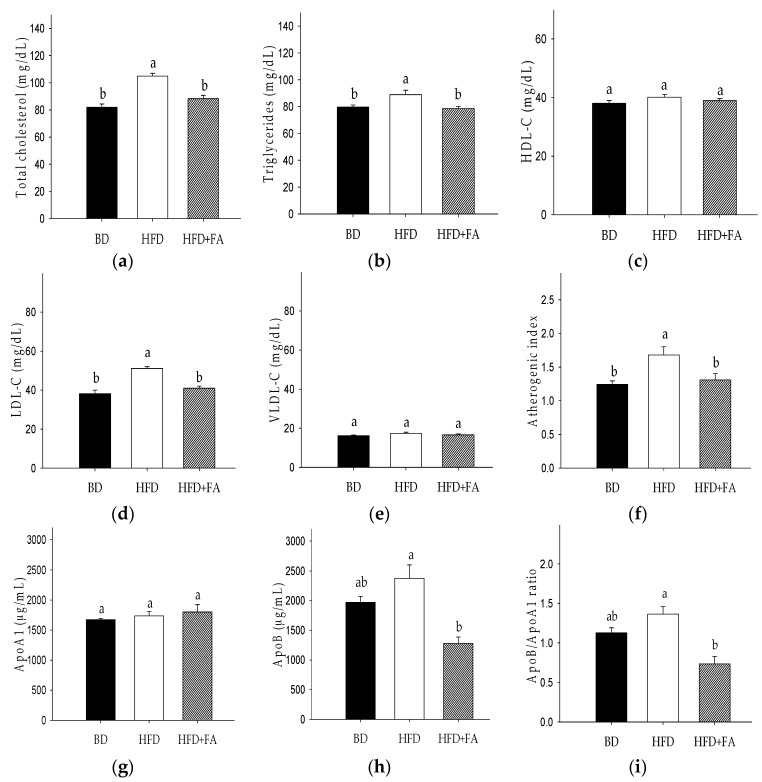
Effect of ferulic acid on the lipid profile in Wistar rats fed a high fat diet (HFD). (**a**) Total cholesterol (TC); (**b**) triglycerides (TG); (**c**) high-density lipoprotein-cholesterol (HDL-C); (**d**) low-density lipoprotein-cholesterol (LDL-C); (**e**) very-low-density lipoprotein-cholesterol (VLDL-C); (**f**) atherogenic index; (**g**) apolipoprotein A1 (ApoA1); (**h**) apolipoprotein B (ApoB); (**i**) ApoB/Apo A1 ratio. Each bar represents the mean ± SEM. Different letters in the bars represent significant differences (𝑝 < 0.05) between dietary treatments.

The serum lipid profile of rats is presented in Figure 1. TC was significantly increased (28%) in the HFD group, as compared to the BD group (*p* < 0.05). An increase of LDL-C (34%) was also observed in the HFD group versus the BD group. An increase of TG in HFD fed rats versus the BD group was 12% (*p* < 0.05). The HFD + FA diet prevented the increase of the lipids versus HFD by 16% (TC), 20% (LDL-C), and 12% (TG). The HFD + FA diet improved the atherogenic index (AI) by approximately 22%, although significant differences were not observed (*p* > 0.05). No significant changes in HDL-C concentration were registered.

HFD–fed rats showed 1.2-fold more ApoB compared with BD-fed rats, whereas that HFD + FA-fed rats showed twofold decreases in the serum ApoB level with respect to HFD-fed rats (Figure 1).

Positive Pearson’s correlations were observed between weight abdominal fat tissue and atherogenic index (*r* = 0.8168, *p* = 0.0012), total cholesterol (*r* = 0.5949, *p* = 0.0248), LDL-C (*r* = 0.6082, *p* = 0.021), and VLDL-C (*r* = 0.6822, *p* = 0.0072) (Table 3).

### 3.3. Effect of Ferulic Acid on Biomarkers of Insulin Sensitivity

**Figure 2:**
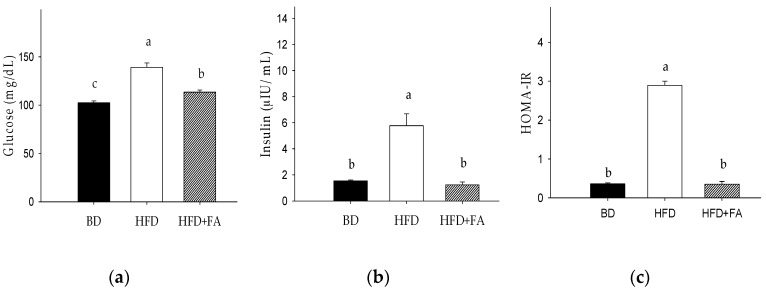
Effect of ferulic acid on biomarkers of glucose metabolism in Wistar rats fed a basal diet (BD), high-fat diet (HFD), and high-fat diet with ferulic acid (HFD + FA) for 60 days. (**a**) Glucose; (**b**) Insulin; (**c**) Homeostatic model assessment index of insulin resistance (HOMA-IR). Each bar represents the mean ± SEM. Different letters in the bars represent significant differences (𝑝 < 0.05) between dietary treatments.

Figure 2 shows the effect of BD, HFD, and HFD + FA on rat blood glucose and insulin levels and HOMA-IR. Blood glucose and insulin levels in the HFD group were significantly higher than those in the BD group (*p* < 0.05). The addition of FA to the high-fat diet significantly reduced blood glucose and insulin levels (Figure 2a,b). As shown in Figure 2c, the HOMA-IR value was eight-fold higher in the HFD compared with BD–fed rats, but this increase was reversed by 0.2% FA supplementation in the HFD. The glucose levels showed a positive correlation with insulin levels (*r* = 0.7695, *p* < 0.0001) and the HOMA-IR (*r* = 0.8672, *p* < 0.0001). Furthermore, positive correlations between weight, abdominal fat tissue, and glucose (*r* = 0.7159, *p* = 0.0027) and HOMA-IR (*r* = 0.85, *p* = 0.0320) were observed (Table 3).

### 3.4. Effect of Ferulic Acid on Inflammatory Biomarkers

**Figure 3:**
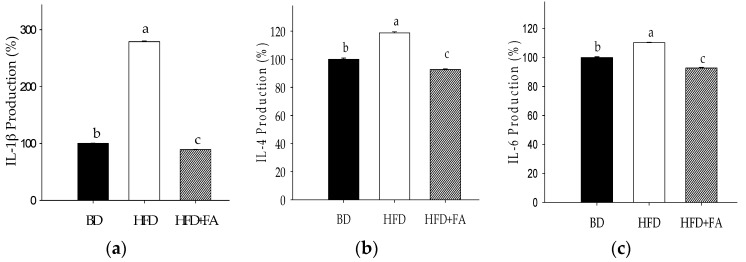

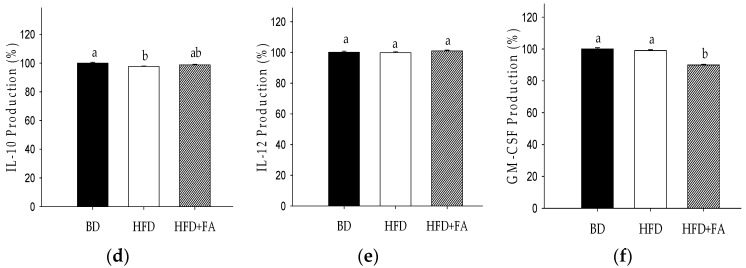
Production (%) of cytokines in plasma of rats fed a HFD and HFD + FA after 60 days of treatment compared to BD poduction (100%). (**a**) Interleukin-1β (IL-1β); (**b**) interleukin-4 (IL-4); (**c**) interleukin-6 (IL-6); (**d**) interleukin-10 (IL-10); (**e**) interleukin-12 (IL-12); (**f**) granulocyte macrophage colony stimulating factor (GM-CSF). Each bar represents the mean ± SEM. Different letters in the bars represent significant differences (*p* < 0.05) between dietary treatments.

An assessment of the major cytokines produced after eight weeks of the HFD diet (Figure 3) revealed a significant elevation in the production of IL-1β, IL-4, and IL-6 compared with serum levels of animals in the BD group. The cytokines IL-10 in HFD-fed animals was slightly decreased with respect to BD, whereas IL-12 showed no significant changes among the dietary groups (*p* > 0.05). The production of GM-CSF did not show significant changes between the HFD and BD, but a significant reduction was observed in the HFD + FA group.

Positive Pearson correlations of IL-6 with glucose (*r* = 0.6131, *p* = 0.0019), HOMA index (*r* = 0.9399, *p* = 0.0002), abdominal fat weight (*r* = 0.6953, *p* = 0.0040), TC (*r* = 0.5304, *p* = 0.0134), LDL-C (*r* = 0.5855, *p* = 0.0042), ApoB (*r* = 0.5793, *p* = 0.0299), IL-1β (*r* = 0.9201, *p* < 0.0001), and IL-4 (*r* = 0.9611, *p* < 0.0001) were observed. Positive correlations of IL-1β with IL-4 (*r* = 0.9618, *p* < 0.0001) and abdominal fat weight (*r* = 0.7952, *p* = 0.0004) were observed (Table 3). 

### 3.5. Antioxidant Capacity of Plasma

**Figure 4:**
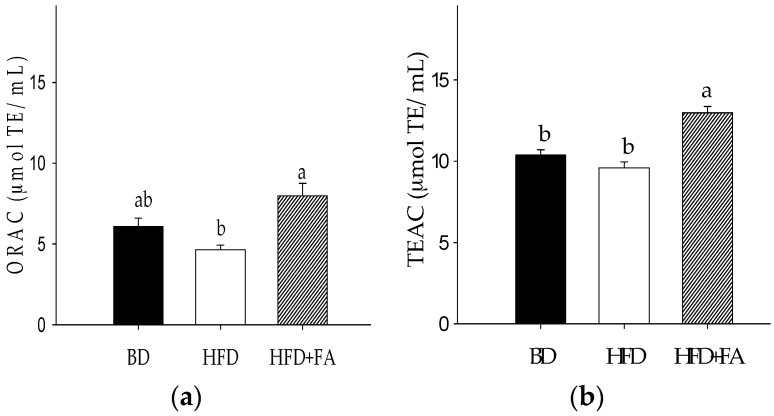
Antioxidant capacity of plasma from rats fed a basal diet (BD), high-fat diet (HFD), and high-fat diet with ferulic acid (HFD + FA) for 60 days. (**a**) Oxygen radical absorbance capacity (ORAC); (**b**) Trolox equivalent antioxidant capacity (TEAC). Each bar represents the mean ± SEM. Different letters in the bars represent significant differences (𝑝 < 0.05) between dietary treatments.

The antioxidant status during obesity and overweight is a key factor that contribute to the initiation and progression of noncommunicable diseases. The plasma antioxidant capacity (ORAC and TEAC assays) in the HFD group was decreased (Figure 4) compared with the BD group rats; however, this decrease was not significant (*p* > 0.05). A significant increase in the plasma antioxidant capacity of the rats in the HFD + FA group compared to the HFD group was found (*p* < 0.05). TEAC and ORAC showed negative Pearson correlations with IL-6 (Table 3).

### 3.6. Histological Study

**Figure 5:**
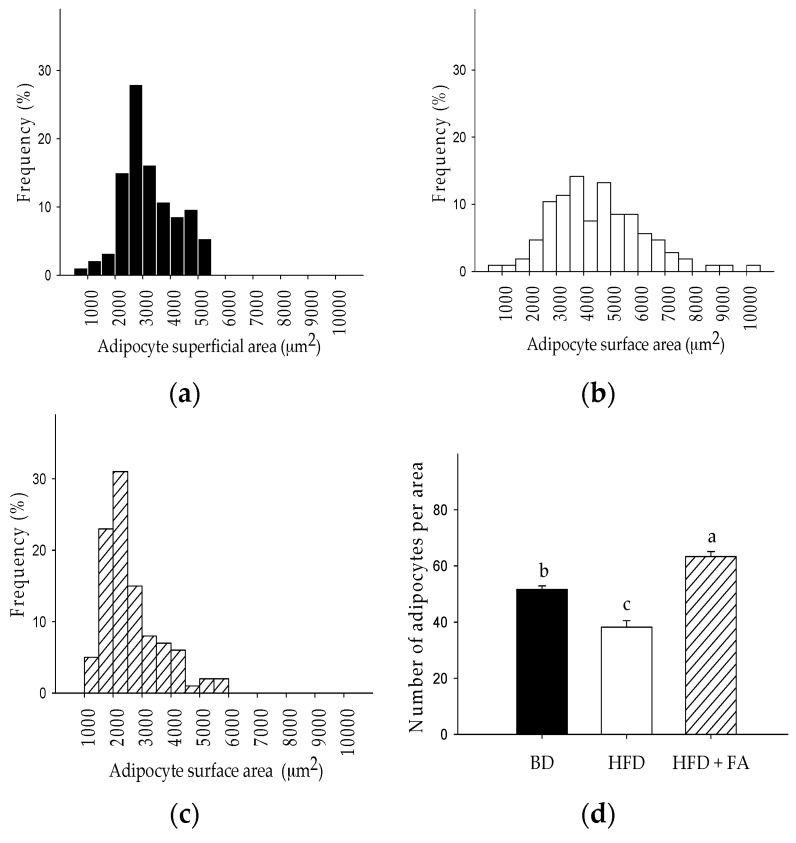
Distribution frequencies of adipocyte size in abdominal adipose tissue of rats were obtained by counting at least 100 cells per subject fed with (**a**) basal diet (BD); (**b**) high-fat diet (HFD); and (**c**) high-fat diet with ferulic acid (HFD + FA) during 60 days; (**d**) Number of adipocytes per area (150,000 μm^2^). The number of adipocytes per area is expressed as the mean ± SEM of five replicates. Different letters on bars represent significant differences (*p* < 0.05) between dietary treatments.

Regarding histological appearance, adipocyte size distribution (superficial area) was larger in the HFD group (500–10,500 μm^2^; mean 4506.10 ± 166.71 μm^2^) than in the BD group (500–5500 μm^2^; mean 3240.4 ± 100.53 μm^2^). However, those in the HFD + FA group were smaller than those in the BD group and the HFD group (Figure 5).

**Figure 6:**
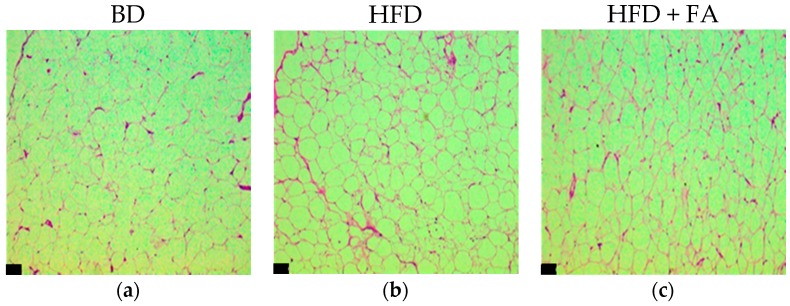

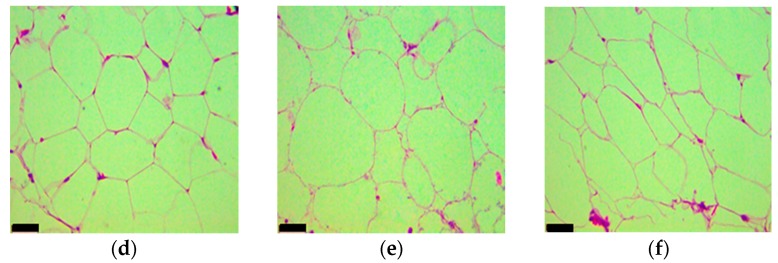
Representative images of adipocytes of abdominal fat tissue of rats fed with a BD, HFD, and HFD + FA during 60 days. The adipocytes were stained with H and E, and the pictures were taken at 10× (**a**–**c**) and 40× (**d**–**f**). Bars on figures represent 34 μm.

The addition of ferulic acid to HFD significantly affected the development of adipocytes. The HFD + FA group had decreased the average size of the adipocytes by 43%, and reduced the size range (1000–6000 μm^2^) compared to rats fed a HFD. The size of the adipocytes was consistent with the results obtained for the number of adipocytes counted per constant area (Figure 5d). In addition to changes in adipocyte size due to intake of the HFD, changes in the hexagonal structure of the normal adipocytes were observed, as shown in Figure 6. The adipocytes of abdominal fat tissue showed a clear hyperplasia (cell number increase) and hypertrophy (cell size increase), which are characteristic of increased fat storage in the adipose tissue.

## 4. Discussion

Obesity and overweight are global health problems, which create an environment conducive to the development of chronic noncommunicable diseases. The study of diseases associated with obesity and overweight, and the search for treatments and strategies for disease control have been the subject of multiple investigations [2,20,21,22,23]. In the present study, the effect of ferulic acid on the abdominal adipose tissue, lipid and glucose homeostasis, inflammation biomarkers, and antioxidant capacity in rats fed with a HFD was investigated.

Ferulic acid showed anti-adipogenic properties because it decreases the weight of abdominal fat tissue, fat index, hyperplasia, and hypertrophy compared to rats fed with a HFD (Table 2). Such results could be due to ferulic acid inhibiting adipocyte differentiation or suppressing lipid accumulation in cells [24] whereby the ferulic acid intake could contribute to the prevention of atherosclerotic cardiovascular disease and its risk factors, which has been associated with abdominal obesity indices [23].

The intake of HFD causes the appearance of typical dyslipidemia characterized by increased content of free fatty acid and triglycerides and, often, increased LDL-C and ApoB with decreased HDL-C [4]. In that aspect, it was observed that ferulic acid supplementation in the HFD reduced the TC and triglycerides levels by 16% and 12%, respectively, compared to the HFD group (Figure 1). In addition, ferulic acid intake decreased ApoB, the ApoB/ApoA1 ratio, and LDL-C, but did not affect HDL-C levels. Therefore, the ferulic acid intake could decrease the coronary heart disease risk associated with overweight and obesity via the reduction of the ApoB/ApoA1 ratio and LDL-C [25]. These results are consistent with previous studies, which showed that ferulic acid has anti-atherogenic properties [26].

Additionally, a positive correlation between inflammatory biomarkers (IL-1β, IL-4, and IL-6) and lipids biomarkers and WAFT (*p* < 0.05) was observed. Previous reports have shown the intake of HFD modified adipocyte normal function with the consequent production of free fatty acids, free radicals and adipokines (TNF-α, IL-6), and monocyte chemoattractant protein-1 (MCP-1). This is a key factor for the development of low-grade inflammation [2,27], which is a determinant factor in lipids and glucose homeostasis [2,28].

In our study, ferulic acid modulated the glucose dyshomeostasis induced by HFD by reducing the glucose, insulin levels, and HOMA-IR (Figure 2), which contribute to the prevention of insulin resistance and type 2 diabetes. In a previous study, ferulic acid was shown to improve lipid and glucose homeostasis by modulating the expression of lipogenic genes (SREBP1c, FAS, ACC), stimulating β-oxidation genes (CPT1a, PPARα), and gluconeogenic enzymes (PEPCK and G6Pase) [29]. Additionally, ferulic acid regulates hepatic GLUT2 gene expression through the modulation of transcription factors, which has been observed in high-fat and fructose-induced type-2 diabetic on adult male rats [30].

On the other hand, HFD + FA hindered inflammatory biomarker production (IL-1β, IL-4, and IL-6). Biomarker IL-6 showed positive correlations with HOMA-IR (*r* = 0.9399) and it was higher than that observed with glucose and insulin (*r* > 0.6). Based on the foregoing, IL-6 production directly affected the glucose metabolism and the HOMA-IR was a major predictor of glucose homeostasis affectation that the individual glucose and insulin measures. Additionally, IL-1β showed to be a key biomarker in the glucose dyshomeostasis, because it showed a positive correlation with glucose, insulin, and HOMA-IR (*r* = 0.9864, *p* < 0.0001). The HOMA-IR index was considered the major predictor of glucose metabolism affectation instead of the individual glucose and insulin measures. Previous research showed IL-1β to be a key factor in the development of insulin resistance and suppression of insulin-induced glucose transport. This can be via the inhibition of insulin-induced phosphorylation of the insulin receptor beta subunit, insulin receptor substrate 1, Akt/protein kinase B, and extracellular regulated kinase [31].

These results indicated that ferulic acid regulated the inflammatory biomarkers induced by HFD, which are determinant factors in lipid and glucose homeostasis. Our results are consistent with previous reports, which showed that ferulic acid has anti-inflammatory properties because of the decrease in the expression of the transcription factor inhibitor of kappa B (IκB), and the increase of the nuclear translocation of the p65 subunit of nuclear factor kappa B (NFκBp65) [10]. Furthermore, ferulic acid may contribute to the modulation of inflammatory processes through suppression of NO production by downregulating the expression of NF-κB-mediated iNOS gene [32].

On the other hand, results showed that HFD + FA increased the antioxidant serum capacity compared to rats fed with a HFD, measured by TEAC and ORAC assays. Additionally, a negative correlation between antioxidant capacity and glucose, insulin, and HOMA-IR was observed showing that the reduction of antioxidant status negatively affected the glucose homeostasis. Similarly, a negative correlation between antioxidant capacity and inflammatory biomarkers (IL-1β, IL-4, and IL-6) was observed. The ferulic acid intake may be beneficial when dyslipidemia and proinflammatory conditions are present since, together, they create an enabling environment for the development of cardiovascular diseases, such as atherosclerosis [4].

**Figure 7:**
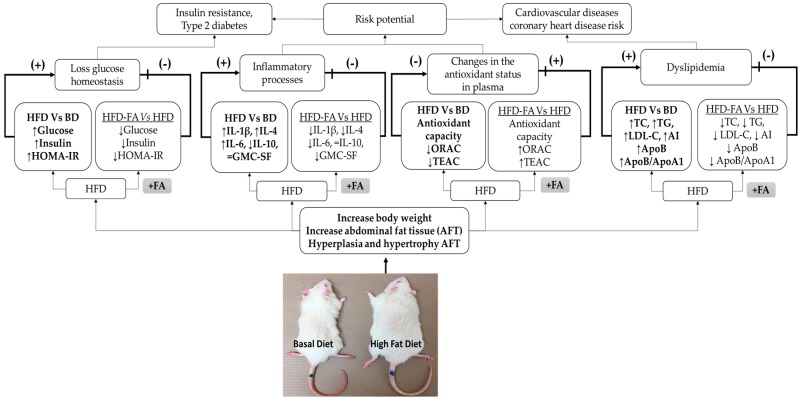
Ferulic acid may contribute to the prevention of the development of chronic noncommunicable diseases induced by the intake of a high-fat diet (HFD). The intake of HFD increased fat storage in adipocytes, consequently increasing the body weight and abdominal fat tissue (AFT), hyperplasia, and hypertrophy in AFT. The factors mentioned above promote dyslipidemia and loss of glucose homeostasis. The modification of adipocytes function causes the release of proinflammatory cytokines which contribute to the generation of chronic low-grade inflammation, which, together with the antioxidant status reduction, are key factors in the development of insulin resistance, type 2 diabetes, cardiovascular diseases, and coronary heart disease risks. The intake of ferulic acid increased the antioxidant status and reduced the proinflammatory cytokines production and storage fat, therefore, ferulic acid may hinder the development of diseases associated with overweight and obesity.

Figure 7 represents a summary of our results, and possible implications are represented. Finally, the results are integrated to show that ferulic acid could be an alternative for prevention of diseases related to dyslipidemia and glucose homeostasis such as diabetes, cardiovascular diseases, and hypertension, which are triggered when the inflammatory processes and oxidative stress are present.

## 5. Conclusions

The results of our study supported our hypothesis since ferulic acid exhibited an integral anti-obesity, anti-inflammatory, and anti-oxidant effects in rats fed a high-fat diet. These results suggest that ferulic acid could be used as a functional and nutraceutical treatment for obesity-related diseases.

## Figures and Tables

**Table 1:** Composition of the experimental diets (g/kg).

	BD ^1^	HFD ^2^	HFD + FA ^3^
Casein	200.0	200.0	200.0
Lard	19	316	316
Oil	24	32	32
Corn starch	501	0.0	0.0
Maltodextrin	118	185	183
Sucrose	0.0	129	129
Methionine	18	18	18
Choline bitartrate	25	25	25
Cellulose	50	50	50
Vitamin mix	10	10	10
Mineral mix	35	35	35
*trans*-Ferulic acid	0.0	0.0	2
Energy (kcal/g)	4.00	6.00	5.98

^1^ BD: basal diet; ^2^ HFD: high-fat diet; ^3^ HFD + FA: high-fat diet supplemented with 0.2% ferulic acid.

**Table 2:** Effect of ferulic acid on food intake, body weight gain, and weight of abdominal fat tissue in rats fed a high-fat diet.

Parameters	BD ^1^	HFD ^2^	HFD + FA ^3^
Food intake (g/day)	12.80 ± 0.44 ^a^	10.85 ± 0.56 ^b^	11.02 ± 0.69 ^b^
Energy Intake (kcal/day)	51.07 ± 1.75 ^b^	65.11 ± 3.38 ^a^	65.91 ± 4.15 ^a^
Food efficiency (g/kcal)	0.022 ± 0.0013 ^b^	0.035 ± 0.0039 ^a^	0.027 ± 0.0029 ^b^
BWG ^4^ (g)	96.50 ± 4.57 ^c^	132 ± 12.59 ^a^	112.15 ± 1.20 ^b^
WAFT ^5^ (g)	5.21 ± 0.97 ^b^	7.77 ± 0.99 ^a^	5.58 ± 0.85 ^b^
Abdominal fat index	1.49 ± 0.27 ^b^	2.07 ± 0.28 ^a^	1.59 ± 0.22 ^b^

^1^ BD: basal diet; ^2^ HFD: high-fat diet; ^3^ HFD + FA: high-fat diet supplemented with 0.2% ferulic acid; ^4^ BWG: body weight gain; ^5^ WAFT: weight of abdominal fat tissue. Values represent the mean ± SEM (*n* = 6). Significant differences between dietary treatments are marked in rows with different letters (*p* < 0.05). The food efficiency (g/kcal) was obtained by body weight gain (g/day)/energy intake (kcal/day) [20].

**Table 3:** Pearson’s correlation for biomarkers of glucose dysregulation, dyslipidemia, inflammation, and antioxidant status in diet-induced obese rats.

	TC	TG	LDL-C	VLDL-C	WAFT	Insulin	IL-1β	IL-4	IL-6	AI	HOMA IR	TEAC	ORAC	ApoB
G	0.5765	0.49	0.6418	-	0.7159	0.7695	0.7668	0.6881	0.6131	0.4945	0.8672	−0.4959	-	-
	(0.0002)	(0.0028)	(<0.0001)		(0.0027)	(<0.0001)	(<0.0001)	(0.0003)	(0.0019)	(0.004)	(<0.0001)	(0.0053)		
TC			0.7947	-	0.5949	-	0.7012	0.5668	0.5304	0.7727	0.5933	-	-	-
			(<0.0001)		(0.0248)		(0.0004)	(0.0074)	(0.0134)	(<0.0001)	(0.0253)			
TG				0.9126	0.765	0.7739	-	-	-	-	0.8083	-	-	-
				(<0.0001)	(0.0014)	(<0.0001)					(0.0005)			
LDL-C					0.6082	-	0.7475	0.6515	0.5855	0.6154	0.7853	-	-	-
					(0.021)		(0.0001)	(0.001)	(0.0042)	(0.0001)	(0.0009)			
VLDL-C					0.6822	0.5003	-	-	-	-	-	-	-	-
					(0.0072)	(0.0049)								
WAFT						-	0.7952	0.7106	0.6953	0.8168	0.85	-	-	-
							(0.0004)	(0.003)	(0.004)	(0.0012)	(0.032)			
Insulin							0.6399	-	0.6214	-	0.994	−0.599	−0.662	-
							(0.0185)		(0.0234)		(<0.0001)	(0.0086)	(0.0099)	
IL-1β								0.9618	0.9201	-	0.9864	−0.6236	−0.6371	-
								(<0.0001)	(<0.0001)		(<0.0001)	(0.0043)	(0.0079)	
IL-4									0.9611	-	0.9623	−0.7269	−0.7278	0.5767
									(<0.0001)		(<0.0001)	(0.0004)	(0.0014)	(0.0308)
IL-6										-	0.9399	−0.7184	−0.7344	0.5793
											(0.0002)	(0.0005)	(0.0012)	(0.0299)
HOMA IR												−0.7755	−0.778	-
												(0.0018)	(0.0081)	

*p*-values are in parenthesis.
